# Emergency Obstetric Care Access Dynamics in Kampala City, Uganda: Analysis of Women’s Self-Reported Care-Seeking Pathways

**DOI:** 10.9745/GHSP-D-24-00242

**Published:** 2024-12-20

**Authors:** Catherine Birabwa, Lenka Beňová, Josefien van Olmen, Aline Semaan, Peter Waiswa, Aduragbemi Banke-Thomas

**Affiliations:** a Department of Health Policy, Planning and Management, Makerere University School of Public Health, Kampala, Uganda.; b Department of Public Health, Institute of Tropical Medicine, Antwerp, Belgium.; c Department of Family Medicine and Population Health, University of Antwerp, Antwerp, Belgium.; d Faculty of Epidemiology and Population Health, London School of Hygiene and Tropical Medicine, London, United Kingdom.; e Global Public Health, Karolinska Institute, Stockholm, Sweden.; f Busoga Health Forum, Jinja, Uganda.

## Abstract

**Introduction:**

Timely access to emergency obstetric care (EmOC) remains a challenge in sub-Saharan Africa, influenced by poor health care utilization and rapid urbanization. Studies show poor maternal health outcomes in African cities, reflecting weak health systems. Understanding care-seeking pathways is key to improving service delivery and health outcomes. We examined self-reported care-seeking pathways among women with obstetric complications in Kampala City, Uganda.

**Methods:**

In this cross-sectional survey, we collected sequential data from 433 women (15–49 years) from 9 health facilities in Kampala City. We developed typologies of common pathways to EmOC and descriptively analyzed key attributes, including median time spent at each step, comparing pathways across complications and participant characteristics. Provider utilization and service delivery performance issues were also assessed.

**Results:**

Participants’ average age was 26 years (standard deviation=6), with 55% (237/433) living outside Kampala. We identified 4 common pathways based on number and location of steps: pathways with 1 step, directly to a facility that provided required care (42%, 183/433); 2 steps, mostly including direct facility referrals (40%, 171/433); 3 steps (14%, 62/433); and 4 or more steps (4%, 17/433). Comprehensive EmOC facilities referred elsewhere 43% (79/184) of women who initially sought care in these facilities. Peripheral facilities referred 65% of women directly to the national referral hospital. A majority (60%, 34/57) of referred women returned home before reaching the final care facility.

**Conclusions:**

Our findings suggest that care pathways of women with obstetric complications in Kampala often involve at least 2 formal providers and reflect possible inefficiencies in the referral process, including potential delays and unnecessary steps. Efforts to strengthen urban health and referral systems should adopt multidisciplinary and integrated approaches, supported by clear policies and structures that facilitate effective interfacility and interdistrict care coordination. This should include streamlined care/referral pathways and equitable emergency transportation systems.

## Abstract (precis)

The findings of this cross-sectional survey suggest that care pathways of women with obstetric complications in Kampala often involve at least 2 formal providers and reflect possible inefficiencies in the referral process, including potential delays and unnecessary steps.

## Teaser

**Key Findings:**

- The pathways to emergency obstetric care (EmOC) were diverse and broadly categorized into 4 major sequences based on the number of steps along an individual’s journey, with a majority of women going directly to a facility that provided the required care.
- Women’s care-seeking pathways reflected potential inefficiencies in the referral system, including referrals from designated comprehensive EmOC facilities and direct referrals to the national referral hospital from lower-level facilities.
- The majority of women seeking care for complications in Kampala resided in neighboring districts, with their pathways often involving 1 or more formal providers before the final care facility. In contrast, women living in Kampala often had more direct pathways.

**Key Implications:**

- Policymakers and program stakeholders should strengthen the urban health system’s capacity for quality EmOC by equitably expanding health care infrastructure, enhancing implementation of referral standards, implementing proven service delivery improvement models, and increasing private-sector contributions.
- Policymakers and programmers should foster collaborative action among metropolitan districts to effectively meet the existing need for quality health care by establishing strong regulatory and accountability mechanisms and coordination systems.

## INTRODUCTION

Over 60% of global maternal deaths occur in sub-Saharan Africa, largely due to treatable obstetric complications, such as postpartum hemorrhage and hypertension in pregnancy.^1^^,^^2^ Timely access to appropriate emergency obstetric care (EmOC) is, therefore, critical for maternal and perinatal survival.^3^ EmOC includes 9 interventions recommended for managing common obstetric complications, with recent proposals suggesting the addition of new quality of care indicators.^4^^,^^5^ Facilities capable of providing 7 of these interventions—administration of parenteral uterotonics, antibiotics and anticonvulsants/antihypertensives, manually removing retained placenta, removing retained products of conception, performing assisted vaginal delivery, and basic neonatal resuscitation—are designated as basic EmOC (BEmOC) facilities. Designated comprehensive EmOC (CEmOC) facilities, in addition to these, are equipped to conduct cesarean deliveries and provide blood transfusions.^5^ High-quality EmOC also requires appropriate referral with appropriate transportation and effective communication between health facilities.^6^ However, timely access to EmOC remains a challenge in many countries in sub-Saharan Africa due to factors, such as poor health service utilization, inadequate household resources, and poor referral practices, and is further exacerbated by ongoing rapid urbanization.^7^^,^^8^

Globally, the population living in cities has increased, estimated at 57% in 2022 and expected to be 68% by 2050.^9^ Africa’s urban population was estimated at 46% in 2022 and is projected to rise to nearly 60% by 2050. While endowed with opportunities for promoting health, cities in Africa face challenges of crowding, with extensive informal settlements, inequality, and inadequate health care infrastructure, which affect access to quality health care and health system performance.^10^^–^^13^ This is reflected by studies that show poor maternal and perinatal health outcomes, such as high institutional maternal mortality and inequitable maternal health services coverage.^14^^,^^15^ The poor outcomes are partly attributed to health care access challenges, such as lack of affordability, inadequate knowledge of where to seek EmOC, and long travel times, coupled with poorly functioning referral systems.^13^^,^^16^^–^^19^ Health outcomes in cities are compounded by surrounding suburban areas, which are shown to have high maternal mortality due to increased travel times.^20^ Understanding care-seeking pathways in urban settings is critical to improving health service delivery and ultimately improving health outcomes.

Understanding care-seeking pathways in urban settings is critical to improving health service delivery and ultimately improving health outcomes.

Studies on health care-seeking in urban settings in Africa show varied patterns including bypassing of primary health facilities for routine care and preference of public health facilities for maternal health services.^17^^,^^21^ Regarding EmOC, particularly, studies have focused on the determinants of access,^7^ with a few studies describing pathways (i.e., series of steps women take along their care-seeking journeys) to care.^22^^–^^24^ Studies in urban settings in sub-Saharan Africa elaborate travel dynamics of women in emergency situations, highlighting different patterns and determinants.^22^^,^^25^^,^^26^ However, these insights emerge from a few cities in Africa, yet women’s care-seeking processes might differ in other cities due to contextual differences in resource availability, governance, and population dynamics, which influence organization of and access to health services.^27^ Additionally, existing studies largely use secondary data from medical records rather than women’s first-hand accounts and provide less information on intermediate steps between place of origin and where women receive care. Insights from women’s lived experiences provide an opportunity to integrate women’s voices in service delivery planning/strategies and improve health system responsiveness.

Uganda has made substantial progress in reducing maternal mortality.^28^ However, poor maternal and perinatal health outcomes in the country’s capital city (Kampala) remain a challenge.^29^ Persistent poor health outcomes amid health care deprivations and a struggling health care system in Kampala present important research areas with potential relevance for other cities in Africa.^29^^,^^30^ In 2020, the Ugandan government prioritized urban areas, with a strategic focus on improving organization of health services and health system performance.^31^ Existing evidence in Kampala on pathways to EmOC describes conditions for and sources of obstetric referrals.^32^^–^^34^ However, this information is limited to a few public health facilities, and there is insufficient evidence on the steps women actually take to reach care. This study aimed to examine and sequentially map self-reported care-seeking pathways among women who had obstetric complications in selected EmOC facilities in Kampala City, Uganda.

## METHODS

### Study Design and Setting

**FIGURE 1:**
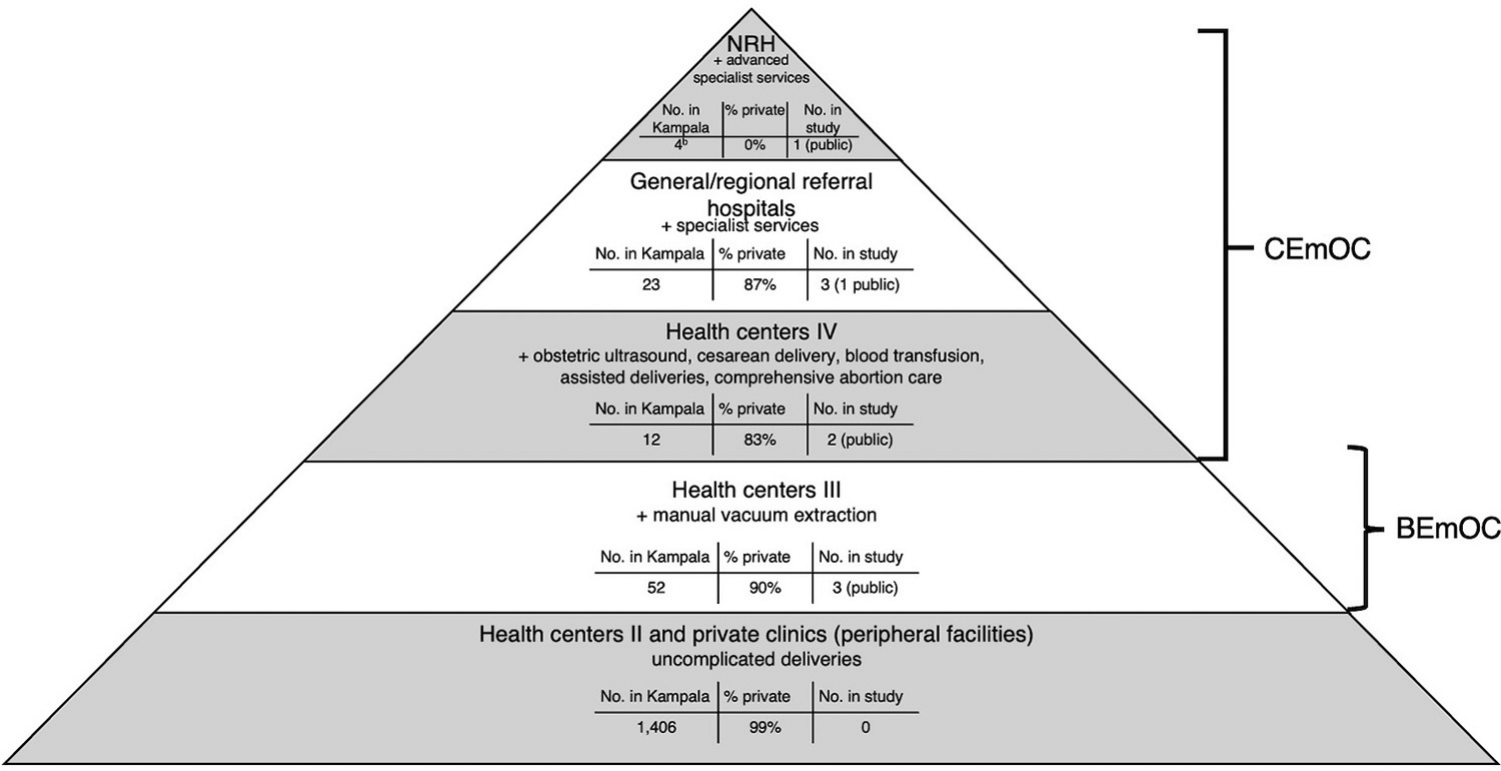
Health Service Delivery Structure^a^ in Kampala, Uganda, for Obstetric Care and Facilities Included in the Study Abbreviations: BEmOC, basic emergency obstetric care; CEmOC, comprehensive emergency obstetric care; NRH, national referral hospital. ^a^Developed for the study based on Uganda’s health service delivery standards.^^33^,^38^^ ^b^Only 1 of the 4 NRHs in Kampala provides obstetric care.

This cross-sectional study was conducted in designated EmOC facilities in Kampala City, Uganda’s oldest and largest urban area, part of the Greater Kampala Metropolitan Area, together with Wakiso, Mukono, and Mpigi districts. Kampala has an estimated resident population of 1.8 million, which swells to 3.5 million during the day.^33^^,^^35^ Kampala exhibits economic disparities, with poverty rates as high as 25% in some parishes.^36^ In 2020, Kampala’s health system, similar to the national health system structure, comprised a mix of 1,497 public and private health facilities,^33^ operating through different levels ranging from health centers (HCs) to the national referral hospital (NRH) (Figure 1). Uncomplicated deliveries can occur at any level, but obstetric complications are managed in CEmOC facilities (HC IV and above).^37^^,^^38^ Although privately owned facilities are the majority (98%, n=1,471), only 2% (n=30) are designated CEmOC facilities. Women predominantly seek obstetric care from the 26 (2%) public facilities that are expected to provide free care. However, household out-of-pocket expenditure on health remains high at 29%, and health insurance coverage is only 2%.^17^^,^^39^

This study was conducted in 9 designated EmOC facilities across the 5 divisions of Kampala City (Figure 1). These included the NRH for obstetrics and gynecology, 5 designated CEmOC facilities, and 3 designated BEmOC facilities. Seven of the facilities, including the NRH, were public, and 2 were private. The facilities were purposively selected to include the typical and major providers of obstetric care in Kampala.^37^^,^^38^ Eight of the facilities are among those conducting the highest number of deliveries in Kampala. In addition to providing advanced specialized services, the NRH provides basic antenatal, delivery, and postnatal care to populations in Kampala and surrounding areas. The hospital’s routine data (2019–2021) showed that it receives more than 10,000 maternity referrals and conducts over 10,000 deliveries per year, of which 42% are cesarean. The 9 facilities include 2 HC IVs, which receive over 1,000 maternity referrals, conduct over 6,000 deliveries, including over 500 cesarean deliveries, and refer over 1,000 maternal cases on average per year. The 2 private hospitals in the sample in a year receive at least 100 maternity referrals, conduct over 2,500 deliveries, including 1,200 cesarean deliveries, and refer 20 maternal cases on average. The 3 BEmOC facilities do not have theaters, conducting over 3,000 deliveries per year on average and referring over 700 maternal cases.

### Study Participants

Women who experienced obstetric complications were approached for inclusion in this study. We included 6 obstetric complications: obstetric hemorrhage (antepartum and postpartum), pre-eclampsia/eclampsia, obstructed/prolonged labor, pregnancy-related sepsis, complications of abortion, and ectopic pregnancy. Women aged 15–49 years who provided written consent to participate in the study were included. Eligible women who declined to or could not participate were not included.

### Sampling and Recruitment

Using a 50% prevalence for pathways to EmOC within which women are referred from 1 facility to another^34^ and a 10% adjustment for non-response, we calculated a sample size of 428 women using the formula for descriptive prevalence studies. We recruited participants from different units/wards within the facilities, including maternity/labor, postnatal, high-dependency unit, gynecology, and emergency departments. We reviewed women’s medical files to identify those with an obstetric complication and confirmed this through consultation with unit/ward in-charge (a midwife or senior nursing officer) or doctors on duty. Participants were sampled consecutively throughout the study period. We aimed to select at least half of the sample size from the NRH due to referral patterns in Kampala.^33^ To ensure variation across the 6 obstetric complications at the NRH, recruitment of obstructed labor cases was stopped as their admissions superseded other complications. In all, we approached 440 women, of which 7 declined to participate.

### Study Variables

Our primary outcome was self-reported care-seeking pathways, retrospectively captured by asking women to detail the steps taken from deciding to seek help for symptoms or going to a health facility for childbirth or routine care up to their final care facility. We used a sequential approach informed by Haenssgen and Ariana^40^ to capture each step’s location, activities, and duration. Locations included different health facilities, informal providers (like traditional birth attendants), and places of self-care/social support (home for the participant or another person). We captured any reported activity done or help received at each step. These included formal care (such as assessment, referral, and delivery), self/informal care (including preparing childbirth items), and ignoring. We captured self-reported time spent in each step and between steps, initially categorized as minutes, hours, days, and weeks, and then quantified. Additional data on transportation, enablers, and barriers faced were collected. Descriptions of pathway attributes and other study variables are provided in Table 1.

**TABLE 1.** Definition of Care-Seeking Pathway Attributes and Other Study Variables, Kampala, Uganda

**Variable**	**Operationalization**
**Care-seeking pathway elements, context, and outcome**	
Location	Places women passed through or were referred from on their pathway to care. The survey captured 10 possible places (locations) where reported activities could have taken place or care was received. For simplicity, we reduced them to: National referral hospital CEmOC facilities (including HC IV and general hospitals) BEmOC facilities Peripheral facilities (including HC II/clinics/medical centers (i.e., including facilities that conduct normal deliveries but are not expected to provide EmOC) Informal providers (including places where detection/identification of an obstetric complication was less likely to happen – drug shop, pharmacy, traditional birth attendant) Self-care/social support (including places where women went to prepare themselves or self-treatment or get items to go with to the health facility, the woman’s home, homes of relatives/friends/neighbors, shops/markets).
Number of steps	Total number of steps in the woman’s pathway. A woman who received care in the first facility where she sought care had 1 step on her pathway.
Activities done/help received	Activities done by the woman or support/care given to her by others she interacts with on her journey. The survey included 10 health and non-health related activities that were grouped as: ignored/did nothing self/informal care (including self-medication, getting care from family/friend, getting permission to seek care, getting transport/funds/requirements, and getting care from a TBA) and formal care (including assessed and referred; stabilized and transferred; delivered the baby; delivered and referred).
Duration (within)	Amount of time spent at each reported location. This was first captured on a nominal scale of minutes, hours, days, and weeks; and then the specific amount of time for each category.
Duration (between)	Time taken between steps. This was first captured on a nominal scale of minutes, hours, days or weeks; and then the specific amount of time for each category of time.
Transportation mode	Transport mode used to move from one location to another. Options included ambulance, private car, public transport (e.g., taxi, bus), special hire, motorcycle, walking, and bicycle.
Companion	The person who was with or helped the woman at the different locations. Options included no one, husband/partner, parent/other relatives, in-laws, friend, community health worker/Village Health Team, traditional birth attendant, and formal health care provider (e.g., midwife, doctor).
Enablers	Factors which made it easy for the woman to do what she did or to get the help she needed at a particular location. This was open text.
Barriers	Factors that made it difficult for the woman to do what she did or to get the help she needed at a particular location. This was open text.
Initial point-of-contact	The first place the woman reported to visit after leaving her place of origin.
Day of the week on which the woman traveled	The day of the week on which the woman traveled to the final facility of care.
Period of the day of travel	The period of the day when the woman traveled to the final facility of care. This was categorized as morning [5 am to 11:59 am], afternoon [12 pm to 4:59pm], evening [5 pm to 8:59 pm], and night [9 pm to 4:59 am].
Waiting time at final facility of care	The amount of time the woman waited before she was attended to at the final facility of care. This was first captured on a nominal scale of attended to immediately, minutes and hours; and then the specific amount of time for minutes and hours.
Reasons for choosing to go to the final facility of care	Among women who intended to go to the (final) facility of care, the reasons why they chose to go to that facility. This was multiple choice and options included: convenience, trust, cost, closes facility, better care, doctors are here, instructed to do so, resources are here, always come here, others
Transportation challenges	The major challenges faced by women regarding transportation to reach the final facility of care. This was multiple choice and categorized as high cost of transport, lack of money for transport, poor access to transport options, inadequate transport options, lack of access to money to use for transport, lack of control over money to use for transport, lack of ambulance, ambulance not functional, traffic jam, others.
**Other study variables**	
Age	Age of the woman in completed years at the time of interview. Categories were also created – <20, 20–24, 25–34, ≥35.
Education	The highest level of education attained by the woman. Categorized as no formal education, primary, secondary (lower S1–S4/higher S5–S6), and post-secondary (including university/diploma/certificate/vocational).
Livelihood	Main source of income for the woman in the past 12 months. Categorized as none (including housewife/student), self-employed, employed by others (including formal employment/traders/etc.), and others.
Marital status	Whether or not the woman was married to or living with a partner. Categorized as in union (including married church/civil/traditional, living with a partner) or not in union (including single/never married, divorced, and separated).
District of residence	The district where the woman lives. Categorized as Kampala, Wakiso, and other districts.
Duration of residence	How long the woman has lived in her current district of residence.
Parity	Number of times the woman had given birth at the time of the interview (as reported by the woman). Categories were created as 0, 1, 2–4, ≥5.
ANC attendance	Whether or not the woman received ANC for the current/recent pregnancy.
Number of ANC visits	If ANC attendance = yes, the number of times the woman attended ANC during the current/recent pregnancy. Categorized as suboptimal (1–3 visits), optimal 1 (4–7 visits), and optimal 2 (≥8 visits).
Fetal outcome	The end result of the pregnancy in relation to the fetus as documented in the woman’s medical record. Categorized as livebirth (full-term), livebirth (preterm), stillbirths, abortive outcomes, unknown/not recorded.
Decision-making capacity	Woman’s involvement in decisions regarding the place of childbirth and transportation (for the current or most recent pregnancy). Categories included woman alone, partner alone, joint between woman and partner, and others (including other household members/people outside the household).
Control over resources	Ability of the woman to use household financial resources independently without asking for permission to support the current EmOC visit.
Freedom of movement	Ability of the woman to travel to seek EmOC without asking for permission.
Referral	A process in which a health care provider at 1 health system level, having inadequate resources to effectively manage a health condition, seeks the assistance of a better or differently resourced facility at the same or higher level to assist in or take over the management of the case.^41^

Abbreviations: ANC, antenatal care; BEmOC, basic emergency obstetric care; CEmOC, comprehensive emergency obstetric care; EmOC, emergency obstetric care; HC, health center; TBA, traditional birth attendant.

### Data Collection

Data were collected using an interviewer-administered questionnaire developed for the study, using KoboCollect.^41^ An excerpt of the questions used to generate the pathway data is provided in (Supplement 1). Nine research assistants, trained and supervised by the lead author, collected the data. These included midwives and graduates with prior experience in facility-based research. We reviewed women’s medical records for information on diagnosis on admission, pregnancy outcome and gestation age. Data were collected between July and September 2022, during the dry season, recognizing that health care accessibility can be affected by and facility choices vary during the rainy season when issues like transportation delays and longer travel times are heightened.^42^^,^^43^

### Data Analysis

Data were cleaned and analyzed in Stata version 14.^44^ Descriptive statistics, including frequencies and percentages, were used to summarize categorical characteristics of participants and pathway attributes; the mean and standard deviation (SD) were used to summarize age across the sample, while the median and inter-quartile range (IQR) were calculated for duration (within/between steps).

We analyzed all individual pathways to identify unique sequences and presented them in a figure. We generated and illustrated a typology of common pathways using the number of steps and their location. At each step, we determined the utilization rates of different sources of care/help as a percentage of women who reported visiting each source. For each common pathway sequence, we summarized relevant attributes from Table 1, considering key obstetric care practices and the urban context, including distribution of demographic characteristics across the pathways. We presented the attributes in tables. During analysis of pathways involving facility referral, we distinguished CEmOC from other health facilities. We described individual pathways of selected women (using pseudonyms) to illustrate common pathway sequences. Next, we explored pathways by complication, analyzing the distribution of common pathways, travel and referral patterns, and how pathways started. We considered 2 possible ways in which women’s care-seeking started: (1) symptoms (a woman experienced something which prompted her to seek care – woman sensed something could be wrong); and (2) labor (a woman went to the facility for routine childbirth care and in the process of or after birth developed the complication; the woman was not aware something could be wrong with the pregnancy when she first sought care). Instances where a woman’s journey started with going to a health facility for routine care, such as ANC, and the complication was detected during this time (signs) were summarized with symptoms. We used chi-square test to compare women’s characteristics by district of residence.

### Ethical Approval

Ethical approval was obtained from the ethics committees of Makerere University School of Public Health (SPH-2021-169), the Uganda National Council for Science and Technology (HS1952ES), the Institute of Tropical Medicine Antwerp (1529/21), and the University of Antwerp Hospital (2021-1743). Written consent to participate in the study was obtained from each participant before the interview. Consent forms were available both in Luganda and English. No directly identifying information, such as individual names and national identification numbers, was collected. Each participant and study site was given a unique identifier.

## RESULTS

### Participant Characteristics

Among the 433 participants, the highest percentage were treated for obstructed labor (32%, 137/433). Participants were aged 26 years on average (SD=6), and the majority were married/cohabiting (79%, 342/433), had secondary education (53%, 227/433), and lived outside Kampala City (55%, 237/433), particularly in Wakiso district (Table 2). More than 76% (335/433) attended ANC, with a maximum of 9 visits. Among all participants, 5% (21/433) experienced a stillbirth. Place of delivery was more commonly decided by women alone (34%, 145/433), while decisions related to transportation (33%, 141/433) and spending money for pregnancy/childbirth care (39%, 169/433) were more frequently made by women’s partners. Further, 33% (141/433) of participants first sought permission to go to the facility to seek care, and this was mostly from their partners (84%, 118/141). Table 2 shows differences in characteristics of participants who lived in Kampala and those from other districts. Parity, requiring permission to spend money, and requiring permission to travel to the facility showed a significant difference.

**TABLE 2.** Characteristics of the Sample of Women Who Experienced Obstetric Complications in Kampala, Uganda

**Characteristic**	**Kampala, % (No.)** **(N=196)**	**Other Districts,**^a^ **% (No.)** **(N=237)**	**Overall, % (No.)** **(N=433)**
Age group, years			
<20	14.3 (28)	8.9 (21)	11.3 (49)
20–24	35.7 (70)	32.1 (76)	33.7 (146)
25–34	39.8 (78)	46.8 (111)	43.7 (189)
≥35	10.2 (20)	12.2 (29)	11.3 (49)
Marital status (n=431)			
Single/divorced/separated	24.2 (47)	17.7 (42)	20.7 (89)
Married/cohabiting	75.8 (147)	82.3 (195)	79.3 (342)
Education level (n=432)			
None/never gone to school	0.5 (1)	1.7 (4)	1.2 (5)
Primary	23.6 (46)	24.9 (59)	24.3 (105)
Secondary	53.9 (105)	51.5 (122)	52.5 (227)
Post-secondary	22.0 (43)	21.9 (52)	22.0 (95)
Livelihood (n=430)			
None (e.g., housewife)/student	35.1 (68)	36.9 (87)	36.0 (155)
Self-employed	22.7 (44)	25.9 (61)	24.4 (105)
Employed by others	34.0 (66)	33.9 (80)	34.0 (146)
Others	8.2 (16)	3.4 (8)	5.6 (24)
ANC attendance			
No	24.5 (48)	21.1 (50)	22.6 (98)
Yes	75.5 (148)	78.9 (187)	77.4 (335)
Number of ANC visits (n=335)			
1–3 (suboptimal)	30.4 (45)	24.6 (46)	27.1 (91)
4–7 (optimal 1)	61.5 (91)	69.0 (129)	65.7 (220)
8–9 (optimal 2)	3.4 (5)	2.7 (5)	3.0 (10)
No response/don’t know	4.7 (7)	3.7 (7)	4.2 (14)
Parity^b^			
0	9.2 (18)	5.1 (12)	6.9 (30)
1	43.9 (86)	36.7 (87)	40.0 (173)
2–4	42.3 (83)	46.0 (109)	44.3 (192)
≥5	4.6 (9)	12.2 (29)	8.8 (38)
Fetal outcome			
Livebirth (full-term)	51.0 (100)	52.1 (123)	51.5 (223)
Livebirth (preterm)	15.3 (30)	18.1 (43)	16.9 (73)
Stillbirth	5.1 (10)	4.6 (11)	4.8 (21)
Abortive outcome	25.5 (50)	21.5 (51)	23.3 (101)
Not recorded	3.1 (6)	3.8 (9)	3.5 (15)
Decision on place of delivery			
Woman alone	33.2 (65)	33.8 (80)	33.5 (145)
Joint decision^c^	25.5 (50)	21.1 (50)	26.6 (115)
Partner alone	21.9 (43)	30.4 (72)	23.1 (100)
Others^d^	16.3 (32)	11.4 (27)	13.6 (59)
No response	3.1 (6)	3.4 (8)	3.2 (14)
Decision on transportation to health facility			
Woman alone	29.6 (58)	24.5 (58)	26.8 (116)
Joint decision^c^	34.2 (67)	31.2 (74)	23.5 (102)
Partner alone	18.9 (37)	27.4 (65)	32.6 (141)
Others^d^	16.3 (32)	16.0 (38)	16.2 (70)
No response	1.0 (2)	0.8 (2)	0.9 (4)
Decision on how to spend money for pregnancy/childbirth care			
Woman alone	21.9 (43)	20.3 (48)	21.1 (91)
Joint decision^c^	41.3 (81)	37.1 (88)	28.6 (124)
Partner alone	25.0 (49)	31.6 (75)	39.0 (169)
Others^d^	10.2 (20)	10.6 (25)	10.4 (45)
No response	1.5 (3)	0.4 (1)	0.9 (4)
Had to request permission to spend money for pregnancy/childbirth care from another person^b^			
No	74.0 (145)	63.7 (151)	68.4 (296)
Yes	26.0 (51)	36.3 (86)	31.6 (137)
Had to request permission to go to the facility^b^			
No	75.0 (147)	61.2 (145)	67.4 (292)
Yes	25.0 (49)	38.8 (92)	32.6 (141)
Complications			
Hemorrhage	15.8 (31)	23.6 (56)	20.1 (87)
Pre-eclampsia	24.5 (48)	24.1 (57)	24.3 (105)
Obstructed labor	31.6 (62)	31.7 (75)	31.6 (137)
Abortion complications	17.4 (34)	6.8 (16)	11.6 (50)
Ectopic pregnancy	9.7 (19)	14.8 (35)	12.5 (54)
Sepsis	6.1 (12)	5.9 (14)	6.0 (26)
How care-seeking started			
Symptoms and signs	(52.0) 102	(50.6) 120	(51.3) 222
Labor	(48.0) 94	(49.4) 117	(48.7) 211

Abbreviations: ANC, antenatal care.

a 84% (199/237) lived in Wakiso district.

b *P*<.05, chi square test.

c Joint between the woman and her partner.

d Others includes other household members, people outside the household, joint decision between woman and other household members.

### Pathways to Care Among Women Who Had Obstetric Complications

We identified 51 unique pathways among the 433 participants (Supplement 2).

#### Initial Point of Contact

Across all pathways, the initial points of contact were as follows: CEmOC facilities (43%, 184/433), peripheral facilities (25%, 110/433), NRH (14%, 60/433), BEmOC facilities (12%, 50/433), self-care/social support (5%, 20/433), and informal care (2%, 9/433). Of the 184 participants who first sought care from a CEmOC facility, 43% (n=79) were referred.

#### Common Pathways to Emergency Obstetric Care

**FIGURE 2:**
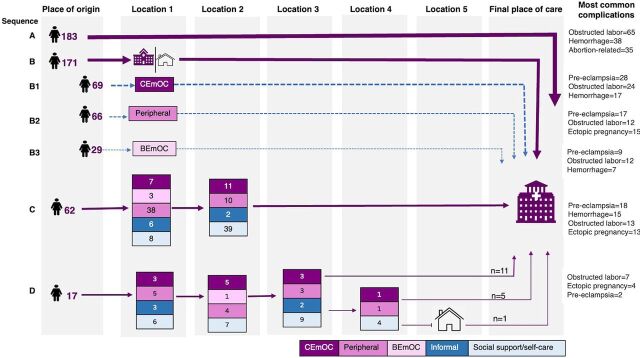
Common Pathways^a^ to Emergency Obstetric Care Among Women From Selected Facilities in Kampala City, Uganda Abbreviations: BEmOC, basic emergency obstetric care; CEmOC, comprehensive emergency obstetric care. ^a^Continuous arrows between the place of origin and “location 1” show the major pathway sequences and the dashed arrows show subsequences.

We grouped the pathways into 4 sequences (A, B, C, and D) based on the total number of steps (ranging from 1 to 6) and location of steps taken (Figure 2). Sequence A, with women who had 1 step on the pathway (direct to the final facility of care), was the most common (42%, 183/433). The most frequent complication in this sequence was obstructed labor (36%, 65/183). Sequence B represents pathways with 2 steps, including the final facility of care (40%, 171/433). The first step was a CEmOC facility (sequence B1) in 40% (69/171), a peripheral facility (sequence B2) in 39% (66/171), a BEmOC facility (sequence B3) in 17% (29/163), and a place of self-care/social support in 4% (7/171, not shown). The most frequent complication in sequence B was pre-eclampsia (32%, 55/171). Sequence C contains pathways with 3 steps, including the final facility of care, and accounted for 14% (62/433) of the sample. The most frequent complication in this sequence was pre-eclampsia (29%, 18/62). Sequence D represents pathways with 4 or more steps, including the final facility of care, representing 4% (17/433) of the sample, with obstructed labor being the most common complication (41%, 7/17).

The most common pathways involved 1 or 2 steps to the final facility of care.

#### Utilization of Sources of Help/Care

Figure 2 shows the distribution of places women visited at each location (step) of the care pathways. Women who did not go directly to final facility (n=250) more commonly first sought care (location 1) from peripheral (44%, n=109) and CEmOC (32%, n=79) facilities. At subsequent steps (locations 2 to 5), places of self-care/social were the most accessed (e.g., 58% at location 2; 46/79).

### Description of Selected Attributes of the Common Pathway Sequences to Emergency Obstetric Care

#### Sequence A (Direct to Final Care Facility)

These were women mostly living in Kampala City (53%), who traveled by motorcycle (54%), seeking care majorly from HC IVs and hospitals where they were treated mostly (55%) for obstructed labor (Table 3). Having someone to help prepare for childbirth was an important facilitator for care-seeking in this group.

**TABLE 3.** Key Attributes of the Common Pathways Among Women^a^ Who Experienced Obstetric Complications in Selected Facilities in Kampala, Uganda

**Pathway Sequence**	**Most Common Transport Mode to Final Facility of Care**	**Median Travel Time to Final Facility, hours (IQR)**	**Main Transport Challenge(s)** ^b^	**Final Facility of Care**	**Main Enabler(s)**	**District of Residence**	**Other Notes**
A: Direct pathways (n=183)	Motorcycle (54%, 99/182)	0.5 (0.5–1)	Financial constraints (51%, 45/88) Traffic jam (44%, 39/88) Inadequate transport options (31%, 27/88)	NRH 33% (n=60) HC IVs and hospitals 57% (n=105) BEmOC facilities 10% (n=18)	Social support from partners, relatives, and friends (51%, 93/183)	Kampala 53% (97/183)	Reasons for choosing facility: Better care (53%, 88/166) Convenient access (31%, 51/166) Usual place of care (26%, 43/166) Common complication is obstructed labor (36%, 65/183). Pathways started with labor in 55% (101/183), and with symptoms/signs in 45% (82/183). 25% (46/183) were attended to immediately, and the rest waited for a median of 0.3 hours (IQR=0.1–0.8).
B1: 2 steps, referral by CEmOC facilities (n=69)	Ambulance (55%, 38/69) Motorcycle (32%, 22/69)	1 (0.5–1.5)	Financial constraints (71%, 27/38) Traffic jam (40%, 15/38) Inadequate transport options (26%,10/38)	NRH (87%, 60/69) Other hospitals (13%, 9/69)	Transport availability (38%, 26/69)	Other districts (65%, 45/69)	Common complication was pre-eclampsia (40.6%, 28/69). Overall, nearly 70% (31/46) of women who had pre-eclampsia and first sought care at a CEmOC facility were referred. Women spent a median of 4 hours (IQR=1–33) in the facility before being referred. 51% were attended to immediately, others (49%) waited for a median of 1 hour (IQR=0.3–2). Care-seeking started with labor in 58% (40/69) and symptoms/signs in 42% (29/69).
B2 and B3: 2 steps, referral by BEmOC or peripheral facilities (n=95)	Motorcycle (39%, 37/95) Ambulance (20%, 19/95)	1 (0.5–1)	Traffic jam (46%, 24/52) Inadequate transport options (42%,22/52) Financial constraints (39%,20/52)	NRH 65% (62/95) HC IVs and hospitals 35% (33/95)	Transport availability (25%, 24/95)	Other districts (62%, 59/95)	Overall, 67% (95/142) of women who first sought care at BEmOC or peripheral facilities went directly to the final facility of care. 48% (46/95) had gone for a check-up because they were not feeling well; while 40% (38/95) had gone to give birth. 28% were treated for pre-eclampsia (27/95) and obstructed labor (25%, 24/95). Women spent a median of 3 hours (IQR=1–8) before being referred; longer in BEmOC (5hours; IQR=1–14) than in peripheral (2hours; IQR=1–7) facilities. 45% (43/95) were attended to immediately at the final facility. Care-seeking started with symptoms/signs in 59% (56/95) and labor in 41% (39/95).
C: 3 steps (n=62)	The first place visited was a peripheral facility in (61%, 38/62). The last place visited before women reached the final facility of care was a place of self-care/social support in (63%, 39/62). 24% (15/62) of women had pathways that included 2 formal providers before the final facility. Between 2 CEmOC facilities (2/15). From peripheral and CEmOC facilities (7/15). Between 2 peripheral facilities (6/15). Among women with pathways where the first step was a formal provider, places of self-care/social support accounted for 60% (34/57) of places visited at the second step. This was highest in pathways of women treated for pre-eclampsia/eclampsia (71%, 12/17) and obstructed labor (70%, 7/10). The most common activity done among the 34 women was obtaining childbirth requirements including money (65% 22/34). Majority lived in other districts (53%, 33/62) and 46% (29/62) lived in Kampala City. 54% (34/62) ended at the NRH and 45% (28/62) ended in HC IVs and hospitals. Care-seeking started with symptoms/signs in 69% (43/62) and labor in 31% (19/62). Nearly 30% (18/62) was among women treated for pre-eclampsia. At the final facility, 25% (16/62) were attended to immediately.						
D: 4 steps (n=17)	All 17 pathways were unique. 35% (6/17) of women had pathways that included more than 2 formal providers before reaching the final facility of care. Majority lived in other districts 65% (11/17). 23% (4/17) ended at the NRH and 77% (13/17) ended in HC IVs and hospitals. Care-seeking started with symptoms/signs in 65% (11/17) and labor in 35% (6/11). Common complication: obstructed labor (41%, n=7) and ectopic pregnancy complications (29%, n=5). 18% (n=3) were attended to immediately at the final facility.						

Abbreviations: BEmOC, basic emergency obstetric care; CEmOC, comprehensive emergency obstetric care; EmOC, emergency obstetric care, HC, health center; IQR, interquartile range, NRH, National Referral Hospital.

a Some data missing in some denominators due to missing answers in the survey.

b Computed among women who reported to have experienced challenges with transportation to the facility; multiple answers possible so totals exceed 100%.

#### Sequence B (One Step Before Final Facility)

Reflecting referral mechanisms in women’s care journeys, this sequence occurred mostly among women who lived in other districts (62.6%, 107/171). The median time spent in the facility before referral was 4 hours (IQR=1–33) in CEmOC, 5 hours (IQR=1–14) in BEmOC, and 2 hours (IQR=1–7) in peripheral facilities. Fifty-five percent of women referred by CEmOC facilities and 20% referred by BEmOC or peripheral facilities used an ambulance for transport to the final facility. Transport availability facilitated care access for at least 20% of participants.

#### Sequence C (Two Steps Before Final Facility)

In pathways of 24% of women, 2 formal health facilities were visited before the final facility. Following initiation of care at a health facility, places of self-care/social support accounted for 60% as the second step. Women with this pathway sequence more commonly lived outside Kampala and were referred by a peripheral facility, with over 50% ending at the NRH.

#### Sequence D (Three and More Steps Before Final Facility)

All 17 pathways had unique sequences, with 6 including more than 1 formal health facility before the final facility. Participants were commonly treated for obstructed labor and lived in other districts. Additional details on each sequence are provided in Supplement 3 and the distribution by participant characteristics in Supplement 4.

### Individual Participant Pathways

**FIGURE 3:**
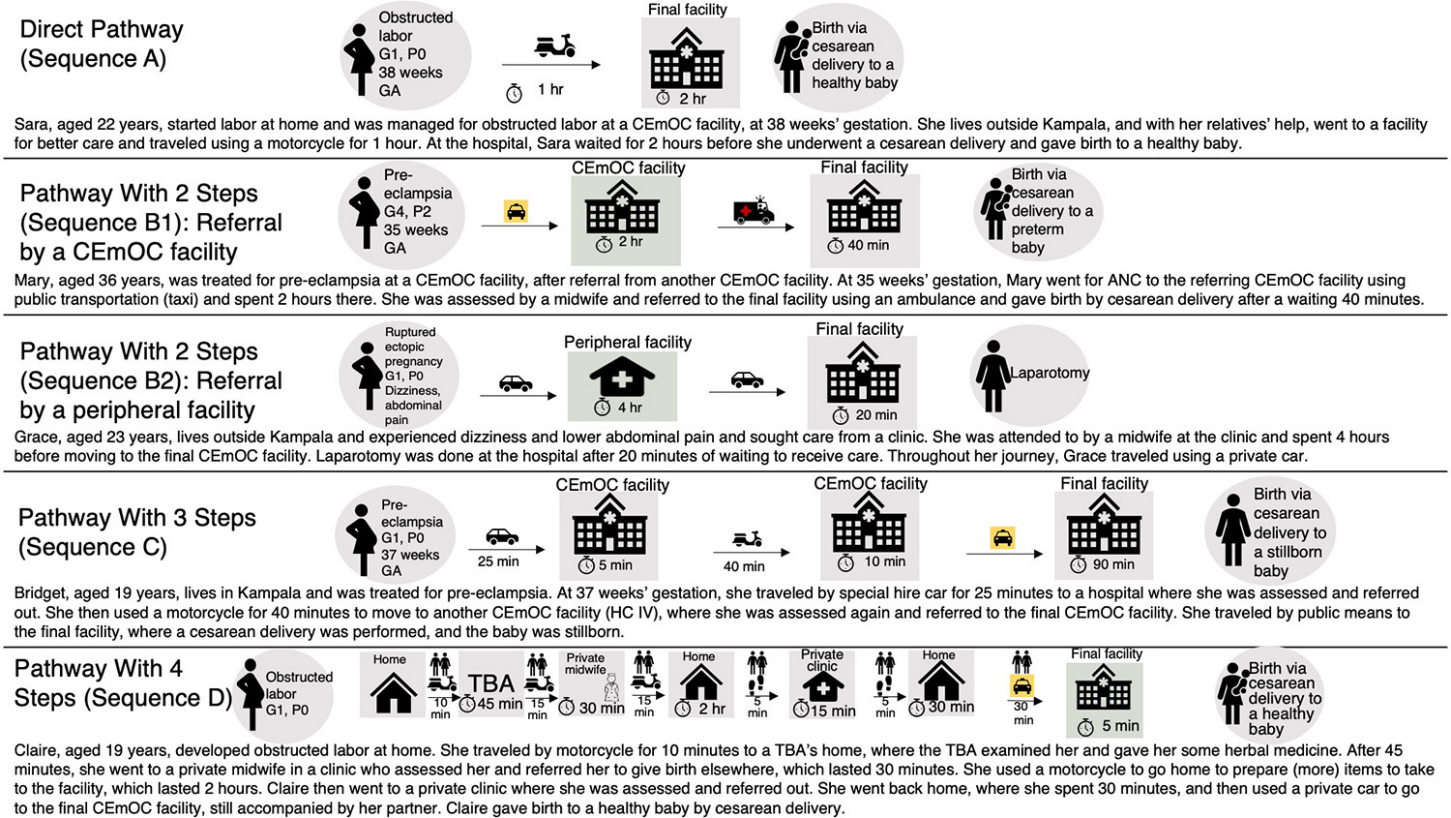
Individual^a^ Pathways Illustrating the Pathway Sequences Abbreviations: ANC, antenatal care; CEmOC, comprehensive emergency obstetric care, G, gravida; GA, gestational age; HC, health center; P, parity, TBA, traditional birth attendant. ^a^All women’s names are pseudonyms.

Illustrations of women’s individual journeys in each sequence described in Table 3 are shown in Figure 3. Starting from the place of origin, the figures show how women with different complications, ages, and obstetric history profiles reached the final facility, and their outcomes. Sequence A and D illustrate the variation in pathways women with the same complication can take to care.

### Description of Pathways by Obstetric Complication

#### How Pathway Started

Pathways among women with sepsis (73%, 19/26), abortion (100%, 50/50), and ectopic pregnancy complications (98%, 53/54) mostly started with symptoms recognized by the woman or signs detected by a health worker (Supplement 5). Among women with hemorrhage (63%, 55/87) and obstructed labor (91%, 125/137), pathways mostly started with women seeking care expecting routine childbirth (labor).

#### Common Sequence

Pathways of women with abortion complications mostly had 1 step (70%, 35/50), while over 50% of pathways of women with all other complications had 2 or more steps.

#### Initial Point of Contact

Over 50% of women across all complications had pathways where the first facility visited was a designated CEmOC facility, except for ectopic pregnancy complications, for which the majority of women first sought care from a BEmOC or peripheral facility (52%, 28/54).

#### Period of Travel

Women more frequently started their journey to the final facility of care in the morning across the 6 complications (168/428, 39%); others traveled in the afternoon (121/428, 28%), evening (68/428, 16%), and night (71/428, 17%).

## DISCUSSION

Our study presents access dynamics for EmOC in Kampala City based on women’s self-reported care-seeking pathways. We found 4 common pathway sequences based on the number and nature of steps taken by participants: (1) direct pathways (42%), (2) pathways with 2 steps (40%), (3) pathways with 3 steps (14%), and (4) pathways with 4 or more steps (4%). These pathways showed that 40% of participants who first sought care at designated CEmOC facilities were referred elsewhere, while 65% of those who first visited BEmOC or peripheral facilities were directly referred to the NRH. Ambulance use was lower during referrals from BEmOC or peripheral facilities (20%) compared to referrals from CEmOC facilities (55%). Additionally, 60% of women returned home after being referred before going to the final care facility.

We found that 4 in 10 participants took a direct path to care. This shows an opportunity to provide timely care with adequate facility readiness. Choice of the facility of care was influenced by perceived quality of care, physical accessibility and familiarity with the health facility, similar to what was found in Eastern Uganda.^45^ Additionally, “early referral” advice given to women with high-risk pregnancies during ANC, as is recommended in Uganda,^37^ could explain direct care-seeking. Our study showed that participants’ social networks played a key role in enabling direct care-seeking, suggesting the importance of involving women’s partners, relatives, and peers in interventions addressing delays in care-seeking. A special focus on vulnerable women lacking social support/capital for childbirth in urban areas is necessary.

The 2-step pathways reflected referral mechanisms and showed that 40% of participants initially seeking care at CEmOC facilities (predominantly HC IVs) were referred elsewhere, notably among women who had pre-eclampsia (70%). This is inconsistent with the expected functionality of CEmOC facilities that should manage nearly all major complications. Moreover, we found that 65% of participants initially seeking care from BEmOC or peripheral facilities were directly referred to the NRH, showing non-adherence to referral guidelines.^37^ These suboptimal referral practices could be attributed to health system constraints, such as a lack of skilled/critical human resources and commodities; inadequate capacity of some CEmOC facilities to manage critically sick patients; or the inability of women to afford care in private hospitals, proximity, and weak enforcement of referral guidelines.^46^^,^^47^ Furthermore, we found that the time women spent before being referred ranged from 2 hours (in peripheral facilities) to 5 hours (in BEmOC facilities). While this time may include prereferral treatment or waiting for transportation, it could also indicate the failure of providers to make timely decisions, which warrants further investigation of quality of care. Strengthening the capacity of health facilities according to their designated levels of functionality and better care coordination are essential to reduce delays from unnecessary referral, bypassing, and crowding at receiving facilities.

The 2-step pathways reflected referral mechanisms and showed that 40% of participants initially seeking care at CEmOC facilities were referred elsewhere.

Our findings showed lower use of ambulances for referrals by BEmOC or peripheral facilities (20%) compared to CEmOC facilities (55%). This indicates that levels of care expected to refer all major obstetric complications, have limited availability or accessibility to ambulance services. In a context of a centralized ambulance coordination system such as in Kampala,^33^^,^^48^ failure of lower-level facilities to use ambulances can arise when multiple requests are made, and priority is given to other types of facilities or patients. Additionally, some peripheral facilities may not be aware of available emergency transportation services and may have limited support to access them. These and other factors related to functionality of ambulances and administrative procedures may explain the apparent low use of ambulances by peripheral facilities. This may translate as additional costs incurred by women as they shoulder the burden of arranging and paying for transportation and exacerbate inequalities in access to EmOC. Improving coverage of affordable ambulance services is crucial to address this issue.

Our results showed that participants tended to prioritize first seeking care at CEmOC facilities, except those who had ectopic pregnancy complications. This behavior reflects women’s expectations for comprehensive care, aligning with service delivery standards that designate CEmOC facilities to manage all major obstetric complications.^37^ This could be a result of women’s understanding of the seriousness of the complications or be influenced by system design. However, we found that nearly a third of participants with 3 or more steps on their path sought care from 2 or more formal health facilities. This reflects the complexity of care-seeking in urban areas and may be influenced by the type or capacity of the health facilities that women visit for EmOC.^23^ However, this finding could also be attributed to women’s preferences, perceived severity of their condition, or unacceptability and unaffordability of the facility to which they were referred.^45^^,^^49^

Our results showed that participants tended to prioritize first seeking care at CEmOC facilities.

Surprisingly, we found that 60% of women referred from 1 facility to another returned home before going to the final care facility. This was common among women with pre-eclampsia (71%) and obstructed labor (70%). This pattern points to inadequate birth preparedness and complication readiness, as women reported that they had to first prepare themselves, get baby clothes, or get money during this time. Poor birth preparedness practices among urban residents have also been reported in Nigeria.^50^ This is likely a result of financial constraints and limited decision-making power of women regarding money for pregnancy/childbirth, which would force women to delay care-seeking.^17^ It could also be attributed to the system’s inadequate support for pregnant women with essential childbirth supplies during their stay in health facilities, as the National Health Insurance Scheme has yet to be rolled out in Uganda, a measure expected to address such challenges.^51^^,^^52^ Inevitably, women bear this burden, which potentially delays their care-seeking.

We found that a majority of the women in our sample seeking care for complications in Kampala lived in neighboring districts or further away. Several factors contribute to this, such as inadequate numbers or readiness of EmOC facilities in neighboring districts, proximity to Kampala that facilitates physical accessibility, and a lack of gatekeeping in Uganda’s health system, enabling individuals to go wherever they want. While Uganda’s decentralization policy mandates districts to manage their population’s health care, there is limited guidance on fostering collaboration across district health systems, a crucial need for the metropolitan context of Kampala. Uganda is currently developing an Urban Health Strategy, which should provide such guidance, including effective referral pathways in metropolitan areas. A potential solution could involve adopting the “networks of care” approach, where health facilities in the metropolitan area are intentionally linked to optimize patient experiences and outcomes.^53^

### Strengths and Limitations

With a large sample of 433 women who had 6 common obstetric complications, our study provides useful insights into different features of pathways to EmOC in an urban setting, which can be used to improve the provision of EmOC services and optimize health outcomes in urban health facilities. We included 8 of the high obstetric volume facilities in Kampala. However, the study also had some limitations. First, due to recruiting from health facilities, the pathways identified may not represent the journeys of women who fail to reach skilled routine or emergency obstetric care. However, given the high facility delivery rate of 94% in Kampala,^54^ the identified pathways provide a good reflection of how women possibly reach care. Also, our recruitment sites included only 2 private hospitals, which might not be representative of all women seeking care in the several private health facilities across the city. Second, we used self-reported data, which could have been affected by response bias. Third, we did not include women who experienced an emergency due to fetal complications and other maternal complications or women who had no complications. Similarly, we did not map pathways of women who experienced maternal death, and some women, particularly those who experienced poor pregnancy outcomes such as stillbirths, declined to participate in the study.

### Implications for Policymakers and Program Managers

#### Strengthen Urban Health System Capacity for Quality Emergency Obstetric Care and Referral

Despite women often seeking care from formal providers, frequent referrals across all facility types reflect system weaknesses. Infrastructure for quality EmOC must be expanded and strengthened, ensuring equitable distribution and accessibility, aligned with actual demand and women’s care-seeking behaviors. This effort should be reinforced with targeted quality improvement programs across all key facility types along women’s care pathways. Additionally, programs are needed to review and enhance the implementation of referral standards and emergency medical services, aiming to reduce inefficiencies and streamline referrals within the metropolitan region. Testing and implementing service delivery models, such as networks of care, the hub-and-spoke model, or localized maternity-neonatal systems, will be essential to optimize the provision of EmOC.^53^^,^^55^ Furthermore, policy initiatives that facilitate access through private hospitals could contribute to improved overall access to EmOC.

Infrastructure for quality EmOC must be expanded and strengthened, ensuring equitable distribution and accessibility, aligned with actual demand and women’s care-seeking behaviors.

#### Foster Inter-District Collaboration

Given the urban population dynamics, where many seeking health care live outside the city, policymakers should support collaborative action among metropolitan district authorities to address pertinent health and service delivery challenges as a region. This requires comprehensive planning, clear guidelines on roles and responsibilities, and robust regulatory and accountability mechanisms.^56^^,^^57^ Service delivery redesign within and across the metropolitan districts may also be crucial.^58^

#### Increase the Affordability of Health Care

About 60% of participants had a need to return home after referral due to financial constraints and personal logistical challenges. To address these barriers, the implementation of the national health insurance scheme may be necessary to improve access to formal care.^59^^,^^60^ However, this requires careful consideration of financing mechanisms, equity, and governance of the insurance scheme.^61^^–^^63^ Additionally, programs are needed to strengthen social support systems and socioeconomic empowerment of vulnerable urban women.

### Implications for Future Research

Our study focused on care-seeking pathways of women with complications. Future studies should integrate assessment of processes and quality of care at the health facilities where women sought care to further understand the alignment between care-seeking and service delivery. Also, future studies should use more objective measures for some pathway attributes, such as time spent within and between different steps, and integrate quality, experience, and cost of care assessment along women’s pathways, as well as women’s preferences and agency for timely access to quality EmOC. We also suggest that future studies compare EmOC care-seeking pathways with those for women who receive routine obstetric care (no complications) and those who experience poor perinatal outcomes, including maternal death, from different levels of care and private health facilities. Such research could identify unique care-seeking differences, shedding light on pathway attributes that contribute to poor outcomes or inequalities in health care access. These studies would evaluate differences in actions taken by women with and without complications while also clarifying the appropriateness—or lack thereof—of choices made by these women at the onset of care-seeking and throughout their journey. Additionally, studies designing and testing models of suitable care and referral pathways or systems in urban settings are necessary.

## CONCLUSIONS

We found 4 common pathway sequences to care in Kampala, with most women going directly to the final care facility. The majority of women referred between health facilities lived in neighboring districts, and direct referral to the NRH was common. Also, most referred women first returned home to prepare themselves before proceeding to the final care facility. Increasing the availability and readiness of CEmOC facilities is crucial to reducing CEmOC-to-CEmOC referrals or bypassing of nearest CEmOC facilities by lower-level facilities. Referral care and health care-seeking competency in Kampala and surrounding districts should be improved at all system levels to achieve effective patient care pathways. The “networks of care” approach could be adopted, which would need capacity-building of peripheral facilities, streamlining and strengthening the quality of facility linkages, and increasing availability of affordable emergency transport options, especially for emergencies originating in peripheral facilities. The development of a national health insurance system that covers the urban poor could greatly improve timely access to care. Future studies should investigate the technical quality and experience of care received in these pathways. Last, women’s social context should be integrated into interventions addressing delays in seeking/reaching EmOC.

## Supplementary Material

GHSP-D-24-00242-Supplement.pdf

## Notes

**Peer Reviewed**

**First Published Online:** December 11, 2024.
