# Supplementary material for: Emergency Obstetric Care Access Dynamics in Kampala City, Uganda: Analysis of Women’s Self-Reported Care-Seeking Pathways
Source: Glob Health Sci Pract. 2024 Dec 20;12(6):e2400242. doi: 10.9745/GHSP-D-24-00242 (PMC11666095; doi:10.9745/GHSP-D-24-00242)

Supplement to: Birabwa C, Beňová L, van Olmen J, Semaan A, Waiswa P, Banke-Thomas A. Emergency obstetric care access dynamics in Kampala city, Uganda: Analysis of women's self-reported care-seeking pathways. *Glob Health Sci Pract*. 2024;12(6):e2400242. https://doi.org/10.9745/GHSP-D-24-00242

SUPPLEMENTS

SUPPLEMENT 1. Questionnaire excerpt used to capture care-seeking pathways.

**Questions used to capture care-seeking pathways among women who had obstetric complications in Kampala city**
I will ask you step-by-step what you did, starting from the moment you 'decided to seek care'. Could you please explain the stages or processes of care that you went through up to when you reached this facility?
a. what did you do first when you noticed you had the symptoms that prompted you or when you decided to go to a health facility?
please specify what other help you got at this step
a.1. What kind of help or treatment did you get at this step?
please specify what other help you got at this step
b. where did this activity take place?
please specify the other place(s) where the activites took place
c. how long did this stage last?
what is the actual number of minutes, hours or days or weeks that the stage/step lasted?
d. Who was with you OR who provided care to you at this step?
If a formal healthcare proivder, ask which cadre of provider? Nurse/midwife, doctor, etc
please specify other attendant(s)
e. What transportation mode did you use to reach this step?
specify other mode of transport
f.1. what made things easy for you to get the help you needed or to do the things you did at this step?
f.2. what made things hard for you to get the help you needed or to do the things you did at this step?
g. How long did it take you reach this step (from the previous)?
what is the actual number of minutes, hours or days or weeks that it took the woman to move to this step from the previous one?
Did you do anything else after this?
h. What transportation mode did you use to get to this facility?
specify other mode of transport
**Additional steps**
i. What happened next?
ii. what kind of help or treatment did you get at this step?
please specify what other activity happened at this step
iii. Where did this (activity) take place?
please specify the other place(s) where the activities took place
iv. How much time did you spend in place?
Please specify actual number of minutes/hours/days/etc?
v. What transport mode did you use to get to this step?
please specify other transport modes used
vi. Who was with you OR who provided care to you at this step?
which type of provider was it? Nurse/midwife, doctor, etc
please specify other attendant(s)
vii. what made things easy for you at this step to get the help you needed or do the things you did?
viii. what made things hard for you at this step to get the help you needed or do the things you did?
ix. How long did it take you reach this step (from the previous)?
what is the actual number of minutes, hours or days or weeks that it took the woman to move to this step from the previous one?
Did you take any additional steps before reaching this particular health facility?
x. What transportation mode did you use to get to this facility?
specify other mode of transport

Supplement to: Birabwa C, Beňová L, van Olmen J, Semaan A, Waiswa P, Banke-Thomas A. Emergency obstetric care access dynamics in Kampala city, Uganda: Analysis of women's self-reported care-seeking pathways. *Glob Health Sci Pract*. 2024;12(6):e2400242. https://doi.org/10.9745/GHSP-D-24-00242

SUPPLEMENT 2. Unique pathway sequences identified across all 433 participants

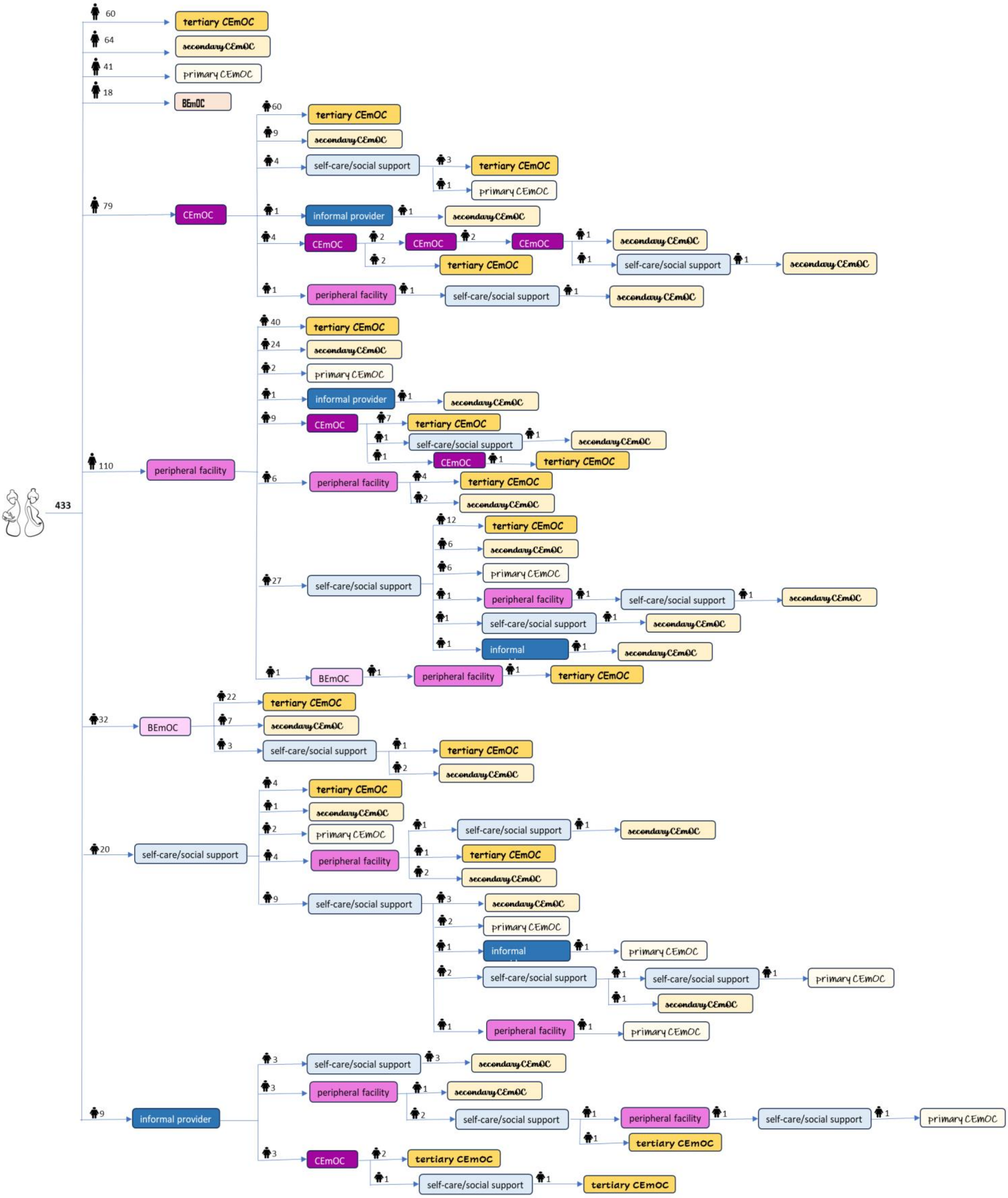

Supplement to: Birabwa C, Beňová L, van Olmen J, Semaan A, Waiswa P, Banke-Thomas A. Emergency obstetric care access dynamics in Kampala city, Uganda: Analysis of women's self-reported care-seeking pathways. *Glob Health Sci Pract*. 2024;12(6):e2400242. https://doi.org/10.9745/GHSP-D-24-00242

SUPPLEMENT 3. Description of the common pathways to EmOC identified among women who had obstetric complications in Kampala (n=433, 9 heath facilities)

Pathway sequence	Key highlights
**A=183**<br>(*direct pathways*)	▪ Care-seeking among women with this pathway started with labour for the majority (55%, 101/183) of whom, 61% (62/101) were treated for obstructed labour.<br>▪ Among the 82 women whose care seeking started with symptoms recognized by the woman, nearly a half were treated for abortion-related complications (43%, 35/82).<br>▪ A majority (53%, 97/183) resided in Kampala, while 43% (79/183) resided in Wakiso and 4%(7/183) lived in other districts.<br>▪ A majority of women travelled on a weekday (81%, 148/183), and 19%(35/183) travelled on a weekend.<br>▪ The most common mode of transport used was the motorcycle (54%, 99/182). Other transport modes were private car (17%, 30/182), public transport (13%, 23/182), special hire (6%, 10/182), ambulance (2%, 4/182) and 3%(5/182) walked.<br>▪ Majority of women reached the final facility of care within one hour (66%, 118/180), and 34%(62/180) travelled for more than one hour to reach the final facility of care<br>○ The longest travel time was three hours, which was among two cases of ectopic pregnancy complications and one case with sepsis.<br>▪ Among women with this pathway, 25%(46/183) were attended to immediately upon arriving at the facility; while 73%(133/183) waited for a median of 0.3 hours (IQR=0.1-0.8)<br>▪ Among the 183 women, 95(52%) did not report any challenges with transportation. Among the remaining 88 women, the top three challenges faced with transport were financial constraints (51%, n=45), traffic jam (44%, n=39) and inadequate transport options (31%, n=27). Pot holes were reported by 7% (n=6) of the women.<br>▪ Of all 183 women, 33%(n=60) went to the NRH; 57%(n=105) went to HC IVs and hospitals facilities; and 10%(n=18)<br>▪ 91% (166/183) of women indicated that they intended to go to the facility where they received care for the complication.<br>○ Reasons for choosing to go to the facility most frequently related to better care (53%, 88/166), convenient access (31%, 51/166) and usual place of care (26%, 43/166). Other reasons given were trust (17%,28/166), cost (11%,18/166), being told to go there by other people (15%, 25/166).<br>▪ Among the 17 women who did not intend to go to the facility where they received care, being in critical condition and being brought to the facility by other people was the most common reason given (41%, n=7). Other reasons were being referred by another facility (24%, n=4) and being told/advised by other people (35%, n=6).<br>▪ Instrumental support in form of having someone (partner, relatives, friends/colleagues) to help pay for care or prepare items to go with to the facility was the most reported enabler to care-seeking (93/183, 51%). Other enablers were availability of transport means (21/183, 12%), having prepared items for childbirth (19/183, 10%), and having money (12/183, 7%).<br>▪ Symptoms experienced by the woman were the most reported barrier (25/183, 14%). For example, being in too much pain and travelling on a motorcycle. Having inadequate money to pay for transport was reported by 6% (11/183).
**B**-*pathways with two steps*	
**B1=69**<br>(referring facility =CEmOC facility)	▪ All referrals were made to hospitals, almost all to the tertiary CEmOC hospital (60/69), where they were treated for pre-eclampsia (28/69) and obstructed labor (24/69).<br>▪ Out of the 69, 54% (n=37) travelled for more than one hour to the facility where they received treatment.<br>▪ Most (55%, 38/69) spent hours within the CEmOC before being referred with a median duration of 4 (IQR=2,6) hours<br>▪ Overall path had a median duration of 5.6 (IQR=2,36.5) hours.<br>○ The shortest path was 0.6 hours (*referred to a secondary CEmOC and was treated for pre-eclampsia*)<br>○ The longest path was 157 hours (*referred to the tertiary CEmOC and was treated for abortion complications*)<br>▪ Among the 69, 31 reported no challenges with transportation to the final facility of care. Of the remaining 38, 71%(n=27) faced financial constraints, 40%(n=15) reported traffic jam, and 26%(n=10) reported inadequate transport options* as challenges.<br>▪ The most frequently reported enabler for getting help at the CEmOC facility was transport availability (38%, 26/69). Others were having money (n=5), social support (n=11), and being worked on immediately (n=4).

	▪ Of the 69 women, 37(54%) did not report any barrier to getting help/care at the CEmOC facility. Less than 10 women reported inadequate money, symptoms and other less frequent (n<5) barriers.<br>Among the 69 women referred directly by a CEmOC facility to the final facility of care:<br>▪ Women spent a median of 4 hours (IQR=1-33) in the first CEmOC facility visited before being referred. The least time spent was among pathways of women treated for pre-eclampsia (2.3 hours; IQR=0.9-7.5), and the longest time was for the one woman who was treated for abortion complications 156 hours (6.5 days).<br>▪ Women travelled for a median of 1 hour (IQR=0.5-1.5). The shortest travel time was among women treated for hemorrhage (0.7 hours), and longest travel time was among women treated for sepsis (3 hours).<br>▪ 51% of women were attended to immediately upon arriving in the final facility of care, and the remaining 49% waited for a median of 1 hour (IQR=0.3-2).<br>▪ Across the six complications, transportation by ambulance was reported by 55% (38/69) of women referred by CEmOC facilities and 32% (22/69) used a motorcycle.<br>o Ambulances were used in at least 30% of pathways of women treated for hemorrhage (32%, 12/38) and pre-eclampsia (37%, 14/38); while motorcycle was commonly used by women treated for obstructed labor (50%, 11/22).
**B2=66**<br>(referring facility =Peripheral facility)	▪ From the peripheral facility, nearly all women (97%, 63/65) were referred to a hospital, more so to the tertiary CEmOC hospital (40/63).<br>o Only two women referred to 1° CEmOC.<br>▪ At least 45% travelled for more than 60 minutes to reach the final place of care (n=31/65), with 42% (27/65) using a motorcycle and 22% (14/65) using a private car.<br>▪ A majority of women reported to have spent hours within the peripheral facility prior to being referred, with a median duration of 3 (IQR=1,3) hours.<br>▪ Overall pathway to final care was over 60 minutes in nearly all women (56/65).<br>o The shortest pathway was 0.5 hours (*referral to a secondary CEmOC and treated for a ruptured uterus*).<br>o The longest path was 87 hours, (*for a woman who spent three days in the peripheral facility before being referred to the tertiary CEmOC hospital where she was treated for ectopic pregnancy complications*).<br>▪ The most common transport challenges faced in reaching final facility of care were traffic jam (53%, 20/38), inadequate transport options (40%, 15/38) and financial constraints (42%, 16/38). Of the 66 women, 28 reported no challenges with transportation.<br>▪ The most frequent enablers to getting care at the peripheral facility were transport availability (20%, 13/66), availability of health workers (17%, 11/66), and having money (15%, 10/66).<br>▪ The most common barriers were the facility lacking capacity to handle the woman's condition (15%. 10/66) and inadequate money (6/66).
**B3=29**<br>(referring facility =BEmOC facility)	▪ All women were referred to hospitals, more so to the tertiary CEmOC facility (22/29, 76%), where they were treated mostly for obstructed labor (12/29)<br>▪ Slightly over 50% travelled within 60 minutes, with 12/29 using an ambulance and 10/29 using a motorcycle.<br>▪ A majority (17/29) reported spending hours in the facility before being referred, with a median duration of 5 (IQR=3,7) hours.<br>▪ The median overall path length was 6 (IQR=2.5,14.3) hours<br>o The shortest path was 0.6 hours (*referral to a secondary CEmOC, treated for pre-eclampsia*)<br>o The longest path was 61 hours (*referral to the tertiary CEmOC, treated for obstructed labor*).<br>▪ Among the 14 women who reported challenges with transportation to the final facility of care, inadequate transport options (n=7, 50%), financial constraints (n=4, 29%) and traffic jam (n=4, 29%) were reported.<br>▪ The most frequently reported enabler to getting care was transport availability (11/29). Others infrequently mentioned were health worker availability (n=3), short waiting time (n=2) and support from social network (n=2)
**C=62**<br>*Pathways with three steps*	▪ The initial point of care was a peripheral facility in 61% (38/62) of pathways.<br>▪ The last point of care before the facility where the complication was treated was a place of self-care or social support in 63% (39/62)<br>▪ 32 unique sequences were identified.<br>▪ Activities related to places of self-care/social support included self-medication, getting care or support from family/friend, getting permission to seek care, getting transport/funds/requirements.
**D=17**	▪ Observed more in women with OL (7/17)<br>▪ Places of formal care were the first point of contact for 47% (7/17)

Supplement to: Birabwa C, Beňová L, van Olmen J, Semaan A, Waiswa P, Banke-Thomas A. Emergency obstetric care access dynamics in Kampala city, Uganda: Analysis of women's self-reported care-seeking pathways. *Glob Health Sci Pract*. 2024;12(6):e2400242. https://doi.org/10.9745/GHSP-D-24-00242

Pathways with four or more steps	▪ All pathways were unique to an individual woman<br>▪ Pathways include intra-facility loops in a CEmOC facility (hospital)<br>▪ The longest pathway (by #steps) shows the woman contacted formal providers at peripheral level twice in between informal and self-care.<br>▪ Most of the pathways point to '*community loops*' – women moving through/between places of non-formal care.

*Includes lack of or unfunctional ambulances.

SUPPLEMENT 4. Characteristics of women who had obstetric complications in Kampala City by pathway sequence

Characteristic	Sequence A (n=183)	Sequence B (n=164)*	Sequence C (n=62)	Sequence D (n=17)
**Age group**				
<20 years	20 (10.9)	19 (11.6)	6 (9.7)	3 (17.7)
20-24 years	59 (32.2)	55 (33.5)	23 (37.1)	5 (29.4)
25-34 years	85 (46.5)	71 (43.3)	27 (43.6)	5 (29.4)
≥35 years	19 (10.4)	19 (11.6)	6 (9.7)	4 (23.5)
**Marital status**				
Single/divorced/separated	43 (23.5)	42 (14.8)	15 (24.2)	5 (29.4)
Married/cohabiting	140 (76.5)	138 (85.2)	47 (75.8)	12 (70.6)
**Education level**				
None/never gone to school	4 (2.2)	1 (0.6)	0 (0.0)	0 (0.0)
Primary	37 (20.3)	42 (25.6)	19 (30.6)	3 (17.7)
Secondary	97 (53.3)	88 (53.7)	30 (48.4)	10 (58.8)
Post-secondary	44 (24.2)	33 (20.1)	13 (21.0)	4 (23.5)
**Livelihood**				
None (e.g., housewife)/student	62 (34.1)	60 (36.8)	20 (32.8)	8 (47.0)
Self-employed	39 (21.4)	46 (28.2)	18 (29.5)	2 (11.8)
Employed by others	72 (39.5)	50 (30.7)	16 (26.2)	6 (35.3)
Others	9 (5.0)	7 (4.3)	7 (11.5)	1 (5.9)
**District of residence**				
Kampala	97 (53.0)	60 (36.6)	29 (46.8)	6 (35.3)
Other districts	86 (47.0)	104 (63.4)	33 (53.2)	11 (64.7)
**ANC attendance**				
No	45 (24.6)	30 (18.3)	18 (29.0)	5 (29.4)
Yes	138 (75.4)	134 (81.7)	44 (71.0)	12 (70.6)
**Parity**				
0	16 (8.7)	10 (6.1)	3 (4.8)	1 (5.9)
1	74 (40.4)	64 (39.0)	23 (37.1)	8 (47.0)
2-4	78 (42.6)	75 (45.7)	30 (48.4)	6 (35.3)
≥5	15 (8.2)	15 (9.2)	6 (9.7)	2 (11.8)
**Fetal outcome**				
Livebirth (full-term)	111 (60.6)	76 (41.5)	25 (40.3)	6 (35.3)
Livebirth (preterm)	19 (10.4)	36 (22.0)	12 (19.4)	4 (23.5)
Stillbirth	6 (3.3)	10 (6.1)	5 (8.1)	0 (0.0)
Abortive outcome	45 (24.6)	32 (19.5)	18 (29.0)	6 (35.3)
Not recorded	2 (1.1)	10 (6.1)	2 (3.2)	1 (5.9)
**Decision on place of delivery**				
Woman alone	74 (40.4)	38 (23.2)	23 (37.1)	5 (29.4)
Joint decision†	39 (21.3)	43 (26.2)	14 (22.6)	4 (23.5)
Partner alone	41 (22.4)	55 (33.5)	13 (21.0)	5 (29.4)
Others*	22 (12.0)	24 (14.6)	10 (16.1)	2 (11.8)
No response	5 (2.7)	4 (2.4)	2 (3.2)	1 (5.9)
**Decision on transportation to the health facility**				
Woman alone	65 (35.5)	29 (17.7)	18 (29.0)	3 (17.7)
Joint decision†	58 (31.7)	56 (34.1)	19 (30.6)	6 (35.3)
Partner alone	33 (18.0)	51 (31.1)	13 (21.0)	4 (23.5)
Others*	25 (13.7)	26 (15.8)	12 (19.4)	4 (23.5)
No response	2 (1.1)	2 (1.2)	0 (0.0)	0 (0.0)
**Decision on how to spend money for pregnancy/childbirth care**				
Woman alone	49 (26.8)	27 (16.5)	12 (19.4)	3 (17.7)
Joint decision†	67 (36.6)	70 (41.7)	26 (41.9)	4 (23.5)
Partner alone	48 (26.2)	50 (30.5)	16 (25.8)	7 (41.2)

Supplement to: Birabwa C, Beňová L, van Olmen J, Semaan A, Waiswa P, Banke-Thomas A. Emergency obstetric care access dynamics in Kampala city, Uganda: Analysis of women's self-reported care-seeking pathways. *Glob Health Sci Pract*. 2024;12(6):e2400242. https://doi.org/10.9745/GHSP-D-24-00242

Others*	17 (9.3)	15 (9.1)	8 (12.9)	3 (17.6)
No response	2 (1.1)	2 (1.2)	0 (0.0)	0 (0.0)
**Had to request permission to spend money for pregnancy/childbirth care from another person**				
No	138 (75.4)	104 (63.4)	37 (59.7)	13 (76.5)
Yes	45 (24.6)	60 (36.6)	25 (40.3)	4 (23.5)
**Had to request permission to go to the facility**				
No	140 (76.5)	92 (56.1)	41 (66.1)	13 (76.5)
Yes	43 (23.5)	72 (43.9)	21 (33.9)	4 (23.5)

*includes only facility pathways, excludes seven whose intermediate step was a place for selfcare/social support

Supplement to: Birabwa C, Beňová L, van Olmen J, Semaan A, Waiswa P, Banke-Thomas A. Emergency obstetric care access dynamics in Kampala city, Uganda: Analysis of women's self-reported care-seeking pathways. *Glob Health Sci Pract*. 2024;12(6):e2400242. https://doi.org/10.9745/GHSP-D-24-00242

SUPPLEMENT 5: Pathways by obstetric complication (n=433)

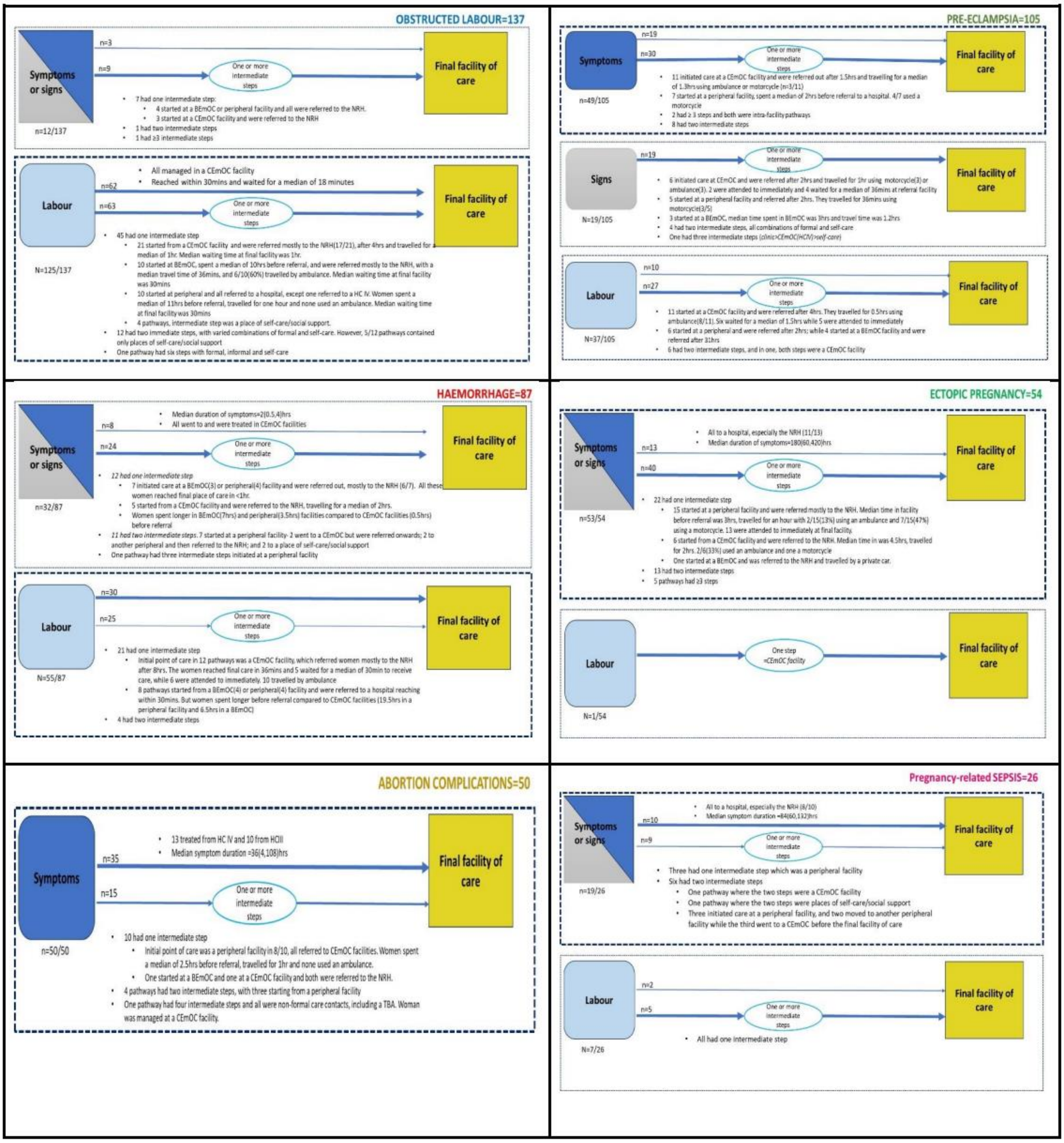

Supplement: GHSP-D-24-00242-Supplement.pdf [file GHSP-D-24-00242-Supplement.pdf]
